# Retraction Note: Copper-based metal–organic framework impedes triple-negative breast cancer metastasis via local estrogen deprivation and platelets blockade

**DOI:** 10.1186/s12951-024-02589-z

**Published:** 2024-05-31

**Authors:** Sijie Wang, Na Yin, Yongjuan Li, Tingting Xiang, Wenxiao Jiang, Xiu Zhao, Wei Liu, Zhenzhong Zhang, Jinjin Shi, Kaixiang Zhang, Xingming Guo, Pilei Si, Junjie Liu

**Affiliations:** 1 School of Pharmaceutical Sciences, Zhengzhou University, Zhengzhou, 450001 China; 2 College of Bioengineering, Chongqing University, Chongqing, People’s Republic of China; 3 Department of Breast Surgery, Henan Provincial People’s Hospital, People’s Hospital of Zhengzhou University, People’s Hospital of Henan University, Zhengzhou, Henan 450003 China; 4 Henan Provincial Engineering Research Center of Breast Cancer Precise Prevention and Treatment, Zhengzhou, Henan 450003 China; 5 Academy of Medical Sciences, Zhengzhou University, Zhengzhou, 450052 China

**Retraction Note: Journal of Nanobiotechnology (2022) 20:313**

10.1186/s12951-022-01520-8

The Editors-in-Chief have retracted this article. After publication, the authors found errors in Figs. 4c, 7h and 7j. Further checks by the publisher found background inconsistencies in the data presented in Figs. 3m and 4c and g and 7c. The Editors-in-Chief therefore no longer have confidence in the presented data.

Junjie Liu has stated on behalf of all authors that they agree to this retraction.

